# Epidemiological Characteristics and Trends of Scarlet Fever in Zhejiang Province of China: Population-Based Surveillance during 2004–2022

**DOI:** 10.1155/2024/6257499

**Published:** 2024-07-14

**Authors:** Zhen Fang, Chenjin Ma, Wangli Xu, Xiuxiu Shi, Shelan Liu

**Affiliations:** ^1^ Center for Applied Statistics School of Statistics Renmin University of China, Beijing 100872, China; ^2^ College of Statistics and Data Science Faculty of Science Beijing University of Technology, Beijing 100124, China; ^3^ The Fourth Medical Center of PLA General Hospital, Beijing 100048, China; ^4^ Department of Infectious Diseases Zhejiang Provincial Center for Disease Control and Prevention, Hangzhou, Zhejiang 310051, China

## Abstract

**Background:**

Over the past two decades, scarlet fever has resurged in some countries or areas. Nationwide nonpharmaceutical interventions changed the patterns of other infectious diseases, but its effects on the spread of scarlet fever were rarely studied. This study aimed to evaluate the changes in scarlet fever incidence in Zhejiang Province, China, before and during the COVID-19 pandemic periods and to provide references for scarlet fever prevention and control.

**Methods:**

Scarlet fever surveillance data in Zhejiang, China (2004–2022), were analyzed in three stages. Two-sample *z* test, ANOVA, and Tukey's test were used to compare and analyze the characteristics of disease spread at different stages. The ARIMA model was used to predict the overall trend. The data were obtained from the National Infectious Disease Reporting Information System.

**Results:**

A total of 28,652 cases of scarlet fever were reported across Zhejiang Province during the study period, with the lowest average monthly incidences in 2020 (0.111/100,000). The predominant areas affected were the northern and central regions of Zhejiang, and all regions of Zhejiang experienced a decrease in incidence in 2020. The steepest decline in incidence in 2020 was found in children aged 0–4 years (67.3% decrease from 23.8/100,000 to 7.8/100,000). The seasonal pattern changed, with peak occurrences in April to June and November to January during 2004–2019 and 2021 and a peak in January in 2020. The median duration from diagnosis to confirmation was highest before COVID-19 (4 days); however, it decreased to 1 day in 2020–2022, matching the other two medians.

**Conclusions:**

In 2020, Zhejiang experienced an unprecedented decrease in scarlet fever, with the lowest incidence in nearly 18 years, but it rebounded in 2021 and 2022. The seasonal epidemiologic characteristics of scarlet fever also changed with the COVID-19 outbreaks. This suggested that nationwide nonpharmaceutical interventions greatly depressed the spread of scarlet fever. With the relaxation of non-pharmaceutical intervention restrictions, scarlet fever may reappear. Government policymakers should prioritize the control of future scarlet fever outbreaks for public health.

## 1. Introduction

Scarlet fever is an acute respiratory infection caused by infectious Group A streptococcus (GAS) bacteria that commonly affects children [1]. Symptoms include sore throat, fever, headaches, swollen lymph nodes, and a rash [2]. Although fatal infections are uncommon, the disease can lead to serious complications such as kidney disease, arthritis, and rheumatic heart disease [3, 4]. Scarlet fever spreads through close contact, via respiratory droplets, or by fomites [5]. As a result, gathering places such as kindergartens, schools, and factories are more likely to experience outbreaks of the fever [6–9].

During the 19th and early 20th centuries, scarlet fever was one of the most feared infectious diseases and a leading cause of child mortality. Like many infectious diseases, its incidence and mortality declined with the advent of antibiotics and the development of economics and healthcare [10–12]. However, in recent years, scarlet fever has reemerged in countries such as the United Kingdom, Vietnam, South Korea, and Australia, due to factors such as the evolution of GAS virulence and the lack of vaccines [7, 13–16]. The upsurge of scarlet fever has also been observed in Mainland China and Hong Kong since 2011 [5, 17].

The COVID-19 pandemic is an ongoing global crisis caused by severe acute respiratory syndrome coronavirus 2 (SARS-CoV-2). Since the first case was reported in Wuhan, Hubei Province, in December 2019, the World Health Organization (WHO) declared it a global pandemic on March 11, 2020 [18]. As of September 25th, 2022, there were 612 million confirmed cases of COVID-19 globally, with 6.5 million deaths reported across more than 220 countries, areas, and territories [19]. In response, most countries took a suppression and mitigation strategy while the Chinese government took a unique nonpharmaceutical interventions (NPIs) to block the spread of this disease, including widespread testing, isolating cases, and social distancing and social distancing measures such as canceling gatherings, issuing stay-at-home orders, limiting travel, and requiring mask-wearing to prevent the spread of the virus [20–22]. Furthermore, these measures were gradually implemented along with the redeployment of healthcare staff to the pandemic response. This resulted in a reduction or reconfiguration of clinical services that provided care for other infectious diseases. More and more evidence suggests that these countermeasures against COVID-19 have been highly effective in preventing multiple infectious diseases, particularly respiratory illnesses [23–26]. However, it remains to be seen whether the upward trend of the scarlet fever will continue into 2020. The resurged countries and the public health community should rally to address this reemerging threat head-on.

Zhejiang Province is situated along the coastal area of East China to the south of the Yangtze River Delta, which has been mildly affected by the COVID-19 pandemic. Since 2011, Zhejiang saw a significant increase in scarlet fever cases, especially in 2015 [4, 27]. In this article, we presented the analysis of Zhejiang provincial surveillance data to assess the COVID-19 pandemic and NPIs' impacts to date on scarlet fever resurgence, stratified by cities and population. This research will help healthcare providers navigate shifting tasks and reset priorities effectively while developing clear guidelines to control the upsurge of scarlet fever after the COVID-19 pandemic.

## 2. Methods

### 2.1. Data Source

In this study, all scarlet fever cases that occurred from January 2005 to December 2022 were obtained from the National Infectious Disease Surveillance System (NNIDSS) [28], which was reported by Zhejiang Provincial Center for Disease Control and Prevention. The criteria for probable, clinical, or laboratory-confirmed cases of scarlet fever were defined according to the Chinese Guidelines for the Diagnosis and Treatment of scarlet fever (2008 Edition) published in December 2008 [12]. All reported cases were reviewed by epidemiologists from local CDCs to ensure the data's completeness and validity.

### 2.2. Data Extraction

We extracted data on scarlet fever, including the number of cases, the incidence of scarlet fever, and patient data on age, sex, and date of disease onset, diagnosis, and death (if applicable). We collected all data available for the study period and no exclusion criteria were used. Zhejiang provincial population data from 2005 to 2022 were obtained from the Zhejiang Provincial Bureau of Statistics.

### 2.3. Statistical Analysis

The yearly and monthly incidences (per 100,000 people) were defined by dividing the number of yearly and monthly cases by the population size. We focused on three timeframes: before (2004–2014) and after (2015–2019) the upsurge of disease and the COVID-19 pandemic stage (2020–2022). The two-tailed two-proportion *z* test was used to compare the monthly incidences in different timeframes. We also computed the growth rate to examine the increase in incidence. For instance, the growth rate of monthly incidence from 2015 to 2019 to 2020 was calculated by the following formula [22]:

(1) $$\begin{array}{c} \frac{\text{monthly incidence in }2020\;-\text{ monthly incidence from }2015\text{ to }2019}{\text{monthly incidence from }2015\text{ to }2019}\times 100\%. \end{array}$$

To identify the trend of monthly incidence before the COVID-19 outbreak, we first used the Cox–Stuart test. Based on monthly incidence in 2005–2019, we fitted the autoregressive integrated moving average (ARIMA) model [29] and predicted the incidence in 2020–2022.

For detailed characteristics, we contrasted incidences of people of different ages, genders, and city groups. We divided the ages into three groups: younger than 15 years old, 15–60 years old, and older than 60 years old. For patients younger than 15 years old, we made a further classification, 0–4 years old, 5–9 years old, and 10–14 years old, to identify the epidemiological patterns. We used ANOVA and Tukey's test to compare the time of cases in three clinical type groups (clinical diagnosed cases, confirmed cases, and suspected cases) in different periods.

All the data analyses were performed using R x64 4.1.2 (Vienna, Austria) and Excel 2019. The ARIMA model was fitted by the function “auto.arima” in the forecast package in R software. The two-tailed two-proportion *z* test was performed by the function “prop.test,” and the ANOVA analysis was performed by the functions “aov” and “TukeyHSD” in the stats package in R software. The significance levels were 0.05 for all tests.

## 3. Results

### 3.1. Overall Yearly Incidence Rate Trend

**Figure 1:**
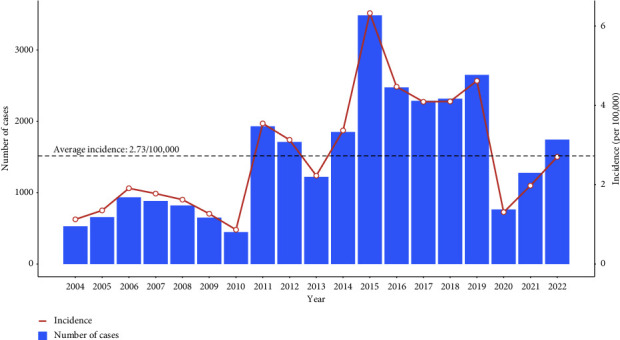
Annual incidence rate and the number of cases of scarlet fever in Zhejiang Province, China, 2004–2022. Note: the average incidence was the mean value of annual incidences from 2004 to 2022.

Between 2004 and 2022, a total of 28,652 scarlet fever cases were reported in Zhejiang Province, with an average yearly incidence of 2.73/100,000. As shown in Figure 1, before 2015, the yearly incidence was almost below the average, with a mean incidence of 2.02/100,000. Then between 2015 and 2019, the mean level rose to 4.72/100,000 and the incidence remained above the average level. After the outbreak of COVID-19, the incidence dropped dramatically, and the figures were 1.31/100,000 in 2020, 1.98/100,000 in 2021, and 2.70/100,000 in 2022, respectively.

### 3.2. Trends and Changes in Monthly Incidence

**Figure 2:**
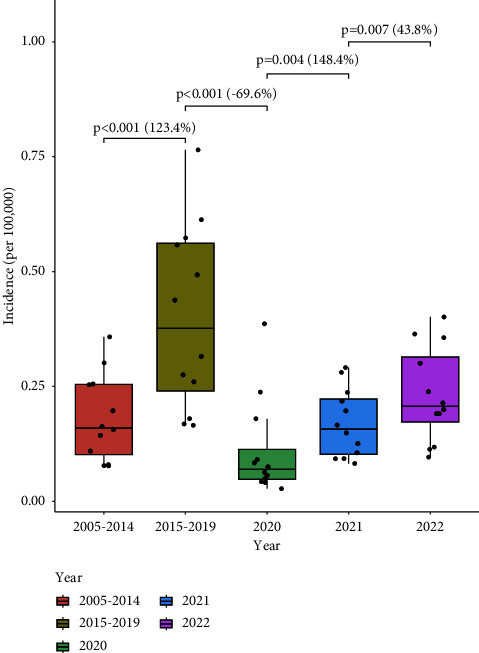
The monthly incidence rates of scarlet fever in 2022, 2021, and 2020 compared with 2005–2014 and 2015–2019 in Zhejiang Province, China. Note: (1) this figure is generated by using the two-proportion *z* test; (2) the black lines in the box indicate P50 from the upper and lower quartiles; the dots indicate the monthly incidence in different years.

The two-proportion *z* test results of the monthly incidence for the three periods and the average growth rates are shown in Figure 2. The monthly incidences in 2020 seemed to be the lowest, and the test results all showed significant differences. In detail, the monthly incidences of 2022 and 2021 were significantly different with *p*=0.007 and growth rate of 43.8%, the monthly incidences of 2021 and 2020 were significantly different with *p*=0.004 and growth rate of 148.4%, the monthly incidences of 2020 and 2015–2019 were significantly different with *p* < 0.001 and growth rate of −69.6%, and the monthly incidences of 2015–2019 and 2004–2014 were significantly different with *p* < 0.001 and growth rate of 123.4%.

### 3.3. Observed and Predicted Monthly Incidence of Scarlet Fever

**Figure 3:**
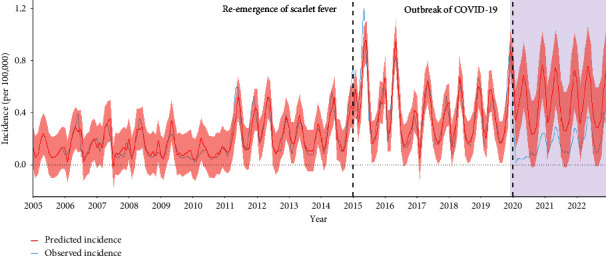
Observed and predicted monthly incidence of scarlet fever by ARIMA model from 2004 to 2022, Zhejiang Province, China.

First of all, the Cox–Stuart test showed an increasing trend (*p* < 0.001) based on 2004–2019 monthly incidences. The fitted model ARIMA(1, 0, 1)(0, 1, 2) [12], as shown in Table 1, was a good fit with RMSE=0.0777 and *R*^2^=0.8513. Based on the above model, the comparison of observed and predicted monthly incidence is shown in Figure 3. The observed monthly incidence after COVID-19 was significantly lower than the predicted value (77.1% lower in 2020, *p* < 0.001; 65.2% lower in 2021, *p* < 0.001; 65.2% lower in 2021, *p* < 0.001; 53.7% lower in 2022, *p* < 0.001).

### 3.4. Trends and Changes in Seasonal Distribution

**Figure 4:**
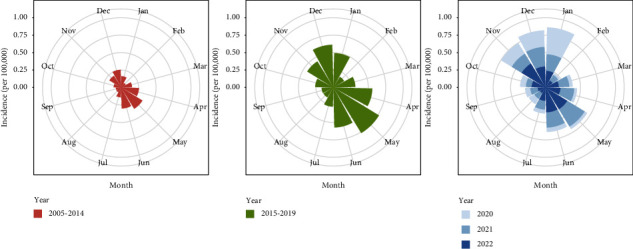
Monthly incidences by three stages: 2005–2014, 2015–2019, and 2020–2022.

As shown in Figure 4, the average monthly incidence was the highest from 2015 to 2019, with the highest incidence up to 0.765/100,000 in May. And the monthly incidence in 2020 seemed to be the smallest (average value: 0.111/100,000). The trends over months followed a similar pattern across 2004–2019, 2021, and 2022. The monthly incidence decreased in February, started to increase in March, peaked in May, and then decreased, with a trough from August to September, and increased slowly in the end. However, the monthly change in 2020 was an exception, with its highest incidence in January (0.386/100,000), followed by a significant decline to 0.0274/100,000 and a rebound to 0.238/100,000 at the end of the year.

### 3.5. Trends and Changes in Age and Gender-Specific Incidence

**Figure 5:**
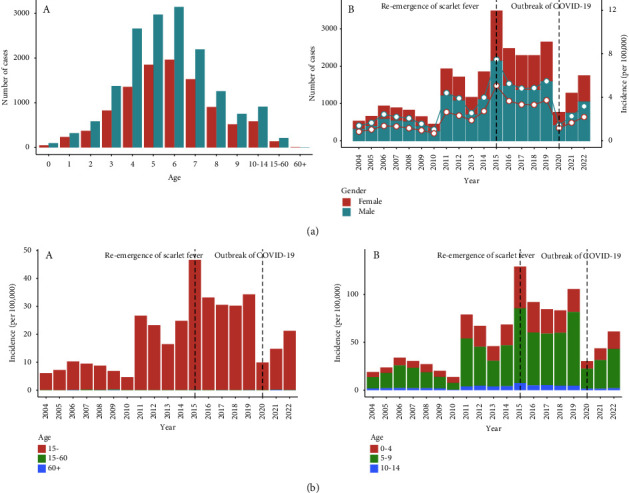
(a) Age and gender distribution of scarlet fever notifications in Zhejiang Province, China, 2004–2022. (A) Total cases by age and gender groups. (B) Annual cases and incidences of males and females from 2004 to 2021. (b) Annual scarlet fever incidences by age groups in Zhejiang Province, China, 2004–2022. (A) Overall age groups. (B) <15 years of age groups.

Figure 5 shows that people under 15 years old were most likely to get infected, accounting for more than 98% of the total cases, while the number of people over 60 years old was close to 0. Among those under 15 years old, children aged 5–9 years old accounted for nearly 75% of the total cases, while babies under 2 years old just took the smallest part. Males (total cases: 17,565, average annual incidence: 3.26/100,000) were more likely to get infected than females (total cases: 11,001, average annual incidence: 2.16/100,000) across all age groups and all years, and the changes in cases and annual incidences of both males and females had the same trends. Especially, in 2015, the number of male cases was 2156 (incidence: 7.47/100,000), while that of females was 1329 (incidence: 5.07/100,000).

Focusing on the annual incidence of people younger than 15 years old, the incidence was relatively low from 2004 to 2010 (less than 10/100,000), and it started rising in 2011 and peaked in 2015 (46.5/100,000). From 2016 to 2019, the incidence fell and remained stable (around 30/100,000). 2020 witnessed a significant reduction to 9.87/100,000, close to the incidence before 2015. And there was a slight increase in 2021 and 2022. Specifically, Table 2 indicates that, after the outbreak of COVID-19, the incidence of children aged under 5 years old dropped fastest with a growth rate of −73.7%.

### 3.6. Trends and Changes in City-Specific Incidence

**Figure 6:**
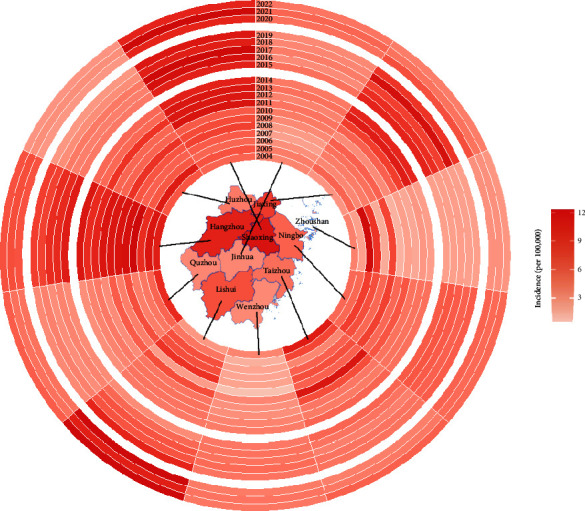
The ring map of scarlet fever yearly incidence by city groups from Zhejiang Province of China, from 2004 to 2021.

The map in the middle of Figure 6 shows the average yearly incidences of cities in Zhejiang Province. The top three cities with the highest mean annual incidence were Shaoxing (5.33/100,000, 5045 cases), Hangzhou (4.66/100,000, 7876 cases), and Jiaxing (3.66/100,000, 3179 cases), with more dramatic changes. These three cities all met a severe scarlet fever outbreak in 2015, and the figures were more than twice the incidences in other cities, with 12.4/100,000 in Hangzhou, 11.7/100,000 in Shaoxing, and 11.4/100,000 in Jiaxing, respectively. However, compared to the average yearly incidence before COVID-19, Huzhou (−93.4%), Taizhou (−72.0%), and Zhoushan (−71.1%) were the top three cities with the smallest growth rates, though the incidences were relatively small. The top three cities with the biggest absolute decrease were still Hangzhou (−3.47/100,000), Jiaxing (−2.44/100,000), and Shaoxing (−2.39/100,000). The incidence in Zhoushan was always the lowest in all the time except 2006 (4.89/100,000). We could conclude that for these cities that met more serious scarlet fever outbreaks, like Hangzhou, Shaoxing, Ningbo, Jiaxing, Wenzhou, Taizhou, and Jinhua, the trends of annual incidence were similar to those shown in Figure 2. However, in Lishui, the incidence started to increase in 2015 and peaked in 2019 (8.69/100,000). And in Huzhou, the incidence peaked in 2012 (4.81/100,000), and then remained low stably.

### 3.7. The Median Days from Onset to Diagnosis among Three Groups

**Figure 7:**
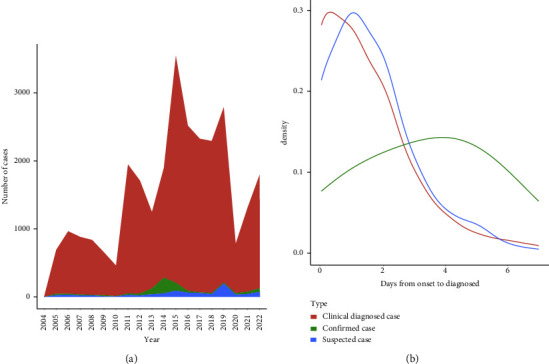
(a) Cases in different clinical type groups from 2004 to 2022. (b) Distribution of days from onset to diagnosis by clinical type groups.

According to Figure 7(a), the change in clinical diagnosed cases, which accounted for 94.5% of all cases, was consistent with the overall trend. The number of confirmed and suspected cases peaked in 2014 (230 cases) and 2019 (196 cases), respectively.  Figure 7(b) was obtained after removing the outliers of time from onset to diagnosis larger than 7 days. Clinically diagnosed cases and suspected cases were similar in terms of days from onset to diagnosed distribution, with the majority of patients diagnosed within 3 days from onset. For these two types of cases, the number of patients declined by the increase of days from onset to diagnosis. While the days from onset to diagnosis of confirmed cases tended to be more equally distributed, there was a slight rise in figures when the days from onset to diagnosis were 4 and 5 days.

Further, ANOVA and Tukey's tests were performed to compare the time from onset to diagnosis in the three clinical groups in three different time stages. The final results shown in Table 3 indicated that the mean of days from onset to confirmation differed significantly from the other two (*p* < 0.001) before COVID-19. However, in the COVID-19 pandemic stage, the days in the three different types of diagnosis showed no significant difference. We calculated the median of days from onset to diagnosis in different time stages, respectively (Table 4). The results showed that the median of days from diagnosed to confirmed was always the highest before COVID-19, but in 2020-2021, the median fell from 4 days to 1 day, equal to the other two medians.

## 4. Discussion

Zhejiang provincial 18-year surveillance data from 11 cities showed a significant decrease in the overall incidence and number of scarlet fever cases during the COVID-19 pandemic in 2020 compared to the pre-COVID-19 period, followed by an increased incidence in 2021 and 2022. The findings of this study suggest that nationwide NPIs may have helped slow down scarlet fever resurgence. However, scarlet fever was likely to rebound in 2021 and 2022 when COVID-19 restrictions were lifted.

Different from the other countries and provinces of China, the incidence of scarlet fever in Zhejiang showed an increasing trend and peaked in 2015 [12, 30, 31]. The accurate mechanisms for the increase are not clear, but some scientists think that some of the incidence increases in scarlet fever may be attributed to the emergence of new genetic lineages of *Streptococcus pyogenes*, the growth of the childish population, and perhaps due to selective pressures such as antimicrobial resistance [4, 12].

Our research showed that the overall scarlet fever incidence rate decreased by 69.6% in 2020 compared to 2015–2019; however, it returned in 2021 and 2022. Several hypotheses have been proposed to explain the substantial decline in 2020. First, because scarlet fever has similar modes of transmission as COVID‐19 (i.e., through respiratory or contact routes), the implementation of public health control measures for COVID‐19 has reduced the frequency of scarlet fever. For example, the government of Zhejiang Province took targeted and intensive measures since the first case was found on January 17th 2020, including the closure and suspension and delayed opening of schools from January to April, canceling big gathering activities, travel restrictions, and regular disinfection [32]. Second, scarlet fever cases were lowly diagnosed due to fewer visits and tests. Third, people's hygiene habits have been greatly improved, including the wearing of masks, hand washing, and the implementation of proper ventilation. Fourth, the use of antibiotics had a great inhibitory effect on the development of scarlet fever in the early period. The incidence in late 2020 to 2022 showed a gradual rebound after the relaxation of public health measures like face masks and social distancing because of the low incidence of COVID-19 in Zhejiang Province. Similar phenomena were reported from outside of Zhejiang, including countries in Europe, East Asia, Oceania, and South America [24, 26, 33–36]. Therefore, it is necessary for governments to pay attention to the recurrence of scarlet fever with the end of the COVID-19 pandemic.

The findings of this study may suggest that nationwide NPIs cannot disrupt the population distribution across age and sex. The age distribution of patients who were reported to have scarlet fever was similar before and after the upsurge and the COVID-19 pandemic. We saw that children aged from 4 to 7 were major victims of scarlet fever. Our age profiling of scarlet fever infections is consistent with those from other countries, with scarlet fever commonly affecting children younger than 10 years [1, 7, 37]. This pattern could be partly attributable to the paucity of herd immunity among children aged 3–6 years to Group A streptococcus infection. Another important reason is that children of this age are attending school for the first time. They may not have developed good hygiene habits yet but need to be in a high-density environment. Additionally, the cases of males were more than those of females [12, 27]. In general, boys were more active than girls and more likely to have improper hygiene, adding to the opportunities for exposure.

The annual incidence of scarlet fever in Zhejiang varied across the 11 cities investigated. The cities with the highest incidence were located in the northern and central regions, with the highest incidence in the north (Shaoxing, Hangzhou, and Jiaxing) and the lowest in Zhoushan. Scarlet fever incidences in all 11 cities decreased greatly in 2020 compared to 2015–2019 but increased in 2021 and 2022 relative to 2020. This difference can be attributed to several factors. Firstly, it is possible that there is a positive association between local incidence and both economy and population density. Cities located in northern and central Zhejiang have well-developed economies and are in close proximity to the cosmopolitan city of Shanghai, which has a higher population density and mobility. Secondly, in terms of topography, cities in northern and central Zhejiang are relatively flat, with mountainous areas in the south. In contrast, Zhoushan has many island districts that are dispersed and relatively less densely populated. Thirdly, the variation in control effects for COVID-19 could partly account for the differences [9, 30, 31]. Additionally, it appears that neighboring cities have an influence on each other.

Consistent with previous reports from China and other regions, scarlet fever incidence is higher in the months of April to June (with a peak in May) and November to January (with a small peak in December) prior to the COVID-19 pandemic [31, 32, 37–40]. The incidence of scarlet fever is influenced by changes in environmental factors such as temperature and humidity and is generally higher in spring and summer than in autumn and winter. During the seasonal changes in May and December, the human body's immune status is more susceptible to the virus. Additionally, scarlet fever's low incidence season coincides with the summer and winter holidays of students, indicating that schools are a crucial site for scarlet fever prevention and control. In 2020, scarlet fever incidence rates followed a different seasonal distribution pattern, with a peak in January and a return to normal afterwards. The activation of the Level I emergency response in Zhejiang Province on 23 January 2020 had a limited impact on the number of new cases reported in January, while the number of cases in the following months remained relatively low under NPIs [32]. The data indicate that the implementation of the nationwide NPIs in China during 2020 had a significant impact on the seasonal distribution of scarlet fever in the country.

The predicted incidence after COVID-19 based on the ARIMA model was higher than the real one. However, 2021 and 2022 witnessed smaller differences between the two values (the observed value was 77.1% lower than the predicted in 2020, 65.2% lower in 2021, and 53.7% lower in 2022). This proved that strict containment strategies during the COVID-19 pandemic had a significant impact on reducing the incidence of scarlet fever. In the long run, with the stabilization of the COVID-19 epidemic and the loosening of interventions, the incidence of scarlet fever tends to rise, calling for the corresponding measures taken by governments.

## 5. Limitations

We analyzed the epidemic characteristics of scarlet fever comprehensively, but there were also some limitations. Firstly, to compare the monthly incidences of different stages, we calculated the average monthly incidences, which might cover the details within single year. Secondly, because of the lack of specific monthly population data, we estimated the monthly incidence by dividing the annual population, potentially leading to a slight miscalculation of monthly incidence. Finally, certain complex factors like individual mental processes were hard to quantify in this study, bringing confounding effects.

## 6. Conclusion

Nationwide NPIshave effectively reduced scarlet fever incidence rates in 2020 and mitigated its resurgence. Although the demographic distribution across age and sex in Zhejiang remained consistent, the seasonal patterns changed in 2020 compared to 2004–2019. Disease patterns were similar across 11 cities. However, scarlet fever incidence increased in 2021 and 2022. With improving NPIs, the scarlet fever will come back again. While most cases are not severe, the hospital admission rate during the upsurge is high, significantly impacting public health. The government should enforce school-based absenteeism and symptom surveillance, establish early warning systems, and allocate additional medical resources to respond to a scarlet fever rebound.

## Figures and Tables

**Table 1:** ARIMA model based on monthly scarlet fever incidence in Zhejiang Province, China.

	AR1	MA1	SMA1	SMA2	Drift
Coefficients	0.6321	0.3360	−0.4625	−0.2303	2 × 10^−3^
Estimation bias	0.0778	0.0916	0.0885	0.0897	7 × 10^−4^
RMSE	0.0060				
*R* square	0.8513				

AR1, MA1, SMA1, and SMA2 represent the first autoregressive coefficient, the first moving average coefficient, the first seasonal moving average coefficient, and the second seasonal moving average coefficient in the ARIMA model, respectively. Drift represents a drift term that captures any trend in the time series data.

**Table 2:** Growth rates (2015–2019 vs. 2020) of annual incidences of scarlet fever among <15 years of age groups, Zhejiang Province, China.

Age	Average incidence in 2015–2019 (per 100,000)	Incidence in 2020 (per 100,000)	Growth rate (%)
0–4	29.7	7.80	−73.7
5–9	63.8	21.0	−67.1
10–14	5.40	1.57	−70.9

**Table 3:** ANOVA and Tukey's test of days from onset to diagnosis by clinical type groups in three stages.

	2005–2014		2015–2019		2020–2022	
	Diff	p. adj	Diff	p. adj	Diff	p. adj
Confirmed case vs. clinically diagnosed case	2.717	<0.001	3.104	<0.001	−0.273	0.987
Suspected case vs. clinically diagnosed case	0.012	0.999	0.358	0.690	−1.029	0.777
Suspected case vs. confirmed case	−2.705	<0.001	−2.746	0.002	−0.757	0.942

Diff represents the difference between the means of the groups being compared, and p. adj represents the adjusted *p* value.

**Table 4:** The median days from onset to diagnosis by clinical type groups in different periods.

	2005–2014	2015–2019	2020-2021
Clinically diagnosed case	1.0	1.0	1.0
Confirmed case	4.0	4.0	1.0
Suspected case	1.5	1.0	1.0

## Data Availability

The datasets supporting the conclusions of this article are available in the National Infectious Disease Surveillance System (NNIDSS) repository (https://www.phsciencedata.cn/Share/ky_sjml.jsp?id=2031b52c-33de-437e-9761-eb4ca2cdfb7b).
